# Celiac Disease and Concomitant Conditions: A Case-based Review

**DOI:** 10.7759/cureus.2143

**Published:** 2018-02-02

**Authors:** Muhammad Uzair Lodhi, Tracy Stammann, Aaron R Kuzel, Intekhab Askari Syed, Rizwan Ishtiaq, Mustafa Rahim

**Affiliations:** 1 Medical Student, Department of Medicine, Raleigh General Hospital, Beckley, Wv; 2 Medical Student, Lincoln Memorial University-Debusk College of Osteopathic Medicine; 3 Department of Emergency Medicine, Lincoln Memorial University-Debusk College of Osteopathic Medicine; 4 Gastroenterology, Beth Israel Deaconess Medical Center, Boston; 5 Assistant Clinical Professor of Internal Medicine, West Virginia University School of Medicine

**Keywords:** celiac disease - type 1 diabetes, celiac disease - rheumatoid arthritis, celiac disease - thyroid problems, celiac disease - inflammatory bowel disease, celiac disease - vitamin deficiencies, celiac disease - lymphoma, celiac disease and sepsis

## Abstract

Celiac disease is a chronic autoimmune disease with genetic predisposition, triggered by the ingestion of gluten. It has a wide range of clinical manifestations ranging from asymptomatic forms to classic presentation of malabsorption with diarrhea and abdominal cramps. Celiac disease can also present with several other concomitant disorders (at the time of diagnosis or during the course of celiac disease) such as: type 1 diabetes, inflammatory bowel disease, rheumatoid arthritis, thyroid disorders, nutritional deficiencies, and gram-negative sepsis. We present a 57-year-old female with past medical history of rheumatoid arthritis, who presented to the emergency department with a complaint of chronic diarrhea, complicated by gram-negative sepsis. The family history of the patient was significant for celiac disease, type 1 diabetes, and rheumatoid arthritis. The patient was closely monitored and treated appropriately. In this case-based review, we explore different associated conditions of celiac disease in the literature, as well as the patient's risk of developing malignancy.

## Introduction and background

Celiac disease (CD) is a common food intolerance, affecting 0.5-1% of the general population [1]. It is a chronic autoimmune disease that affects the mucosa of the small intestine, particularly the duodenum, due to gluten insensitivity. Gluten is found in barley, rye, and wheat. Celiac disease presents with characteristic histopathological findings of blunting of the villi, lymphocytic infiltration, crypt hyperplasia, and distention of the lamina propria. Celiac disease can also be diagnosed with positive serologic testing for anti-transglutaminase antibodies, anti-endomysial antibodies, and anti-gliadin antibodies. It is thought to be associated with many other conditions, particularly autoimmune diseases. The implementation of a gluten-free diet improves the overall clinical course of the celiac disease as well as its concomitant conditions [2]. We hereby present a case of a 57-year-old female with rheumatoid arthritis that presented with complaints of chronic diarrhea. Gram-negative sepsis complicated the clinical course, and the patient was managed appropriately.

Case presentation 

*History and Physical Examination* A 57-year-old female of Eastern European descent presented to the emergency department with chronic diarrhea for the last seven months. The patient reported severe watery diarrhea occurring seven to eight times a day, which turned bloody three weeks ago. It was associated with urgency, abdominal cramping, nocturnal stools, nausea, and weight loss of over 35 lbs in the last seven months. The patient denied any recent travel history or changes in dietary habits. Past medical history of the patient was significant for rheumatoid arthritis (RA) diagnosed six years ago. A detailed review of the family history revealed celiac disease, type 1 diabetes (T1D), and rheumatoid arthritis in her siblings and parents. Upon initial physical examination, the patient appeared to be in mild distress. Her vitals were as follows: blood pressure of 87/59 mmHg, heart rate of 123 beats per minute, respiratory rate of 16 breaths per minute, oxygen saturation of 90% on room air, and fever of 101.8 F. Her body mass index (BMI) was 17. The patient appeared to have intravascular volume depletion with pale, dry skin, and delayed capillary refill of about four seconds. Cardiac auscultation revealed regular rhythm without murmurs or gallops, and audible S1 and S2. Her lung examination was unremarkable. She had tenderness in the epigastric region, and no organomegaly was noted. *Hospital Course *

**Figure 1:**
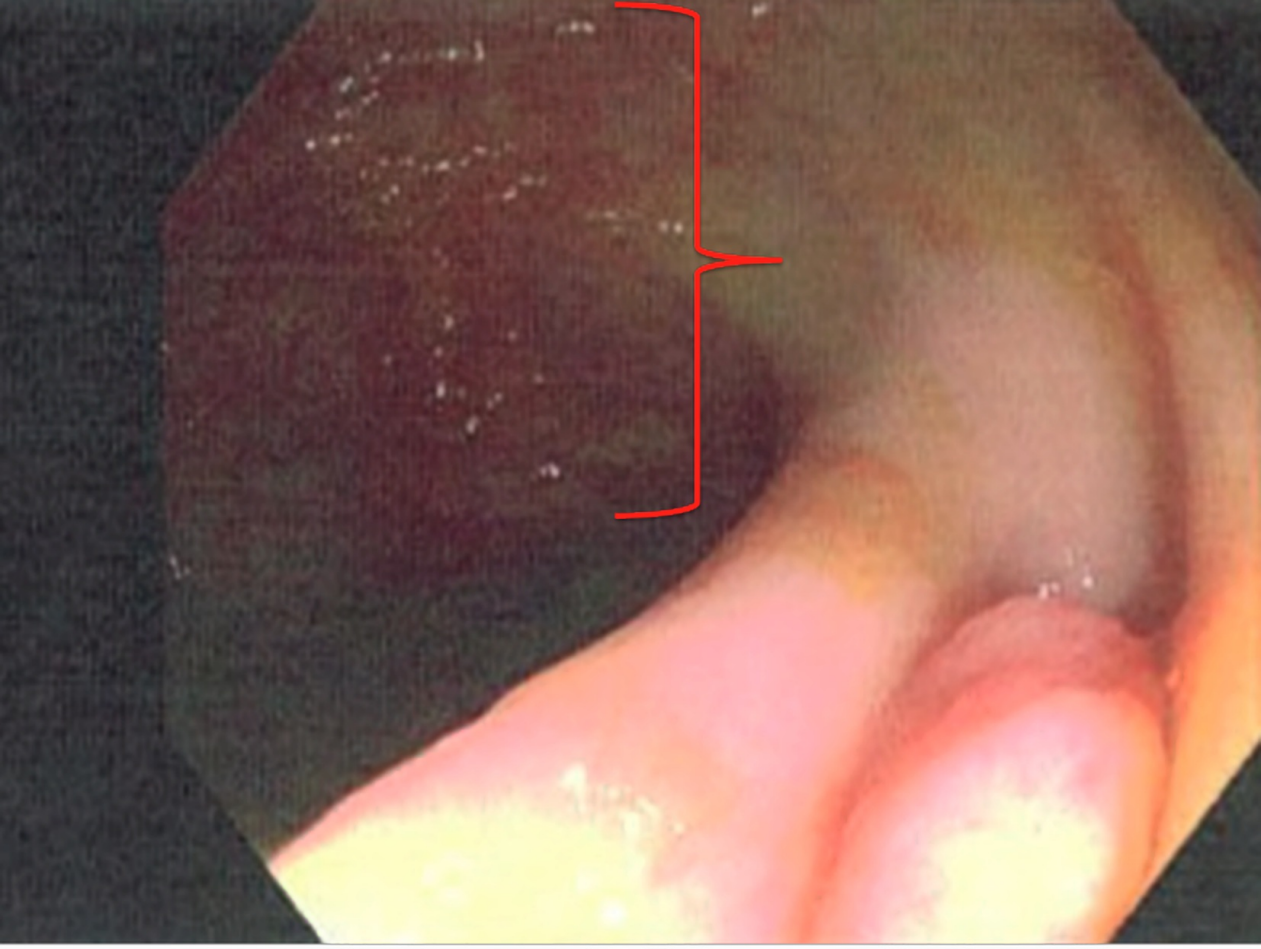
Colonoscopy image showing patchy erythema (red margin) surrounded by normal mucosa near the ileocecal valve.

The patient's serum electrolytes and other hematologic studies were normal except leukocytosis of 13 × 109/L (normal reference range: 4.5 to 11.0 × 109/L) and elevated blood lactate level of 2.5 mmol/L (normal reference range: 0.5-1 mmol/L). The low blood pressure, tachycardia, elevated lactate, and leukocytosis directed us to establish the initial diagnosis of septic shock. The patient's blood cultures were drawn, followed by administration of intravenous fluids and broad-spectrum antibiotics. The patient's vital signs returned to normal within 24 hours, however, the diarrhea persisted. Blood cultures after 48 hours showed growth of enterohaemorrhagic Escherichia coli (EHEC). Stool examination revealed the absence of any positive ova or parasites. Broad-spectrum antibiotics were discontinued, and loperamide (anti-motility agent) was started for symptomatic treatment of diarrhea. Over the next few days, repeated blood cultures showed the absence of any bacterial growth, but the patient still had watery diarrhea occurring six to seven times a day. At this point, further diagnostic studies were considered to find the underlying cause of diarrhea. Colonoscopy showed mild erythema of the colonic mucosa (Figure 1).

**Figure 2:**
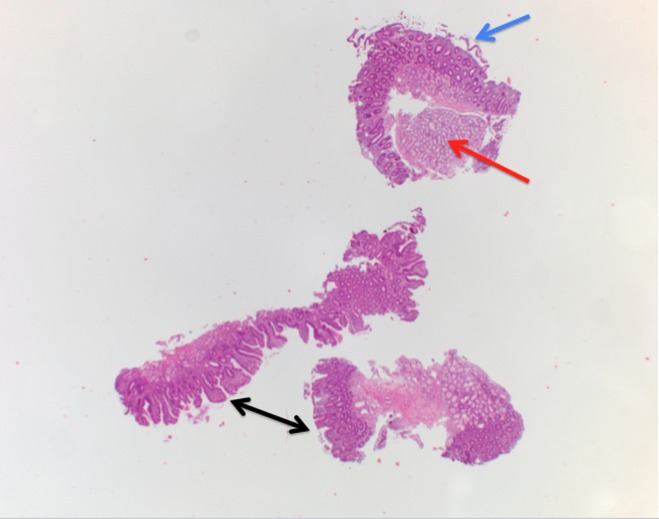
Biopsy image (H&E stain) of the duodenum. Image showing Brunner’s gland hyperplasia (red arrow), complete flattening of the villi (blue arrow), and variable blunting of the villi (black arrows) consistent with celiac disease.

**Figure 3:**
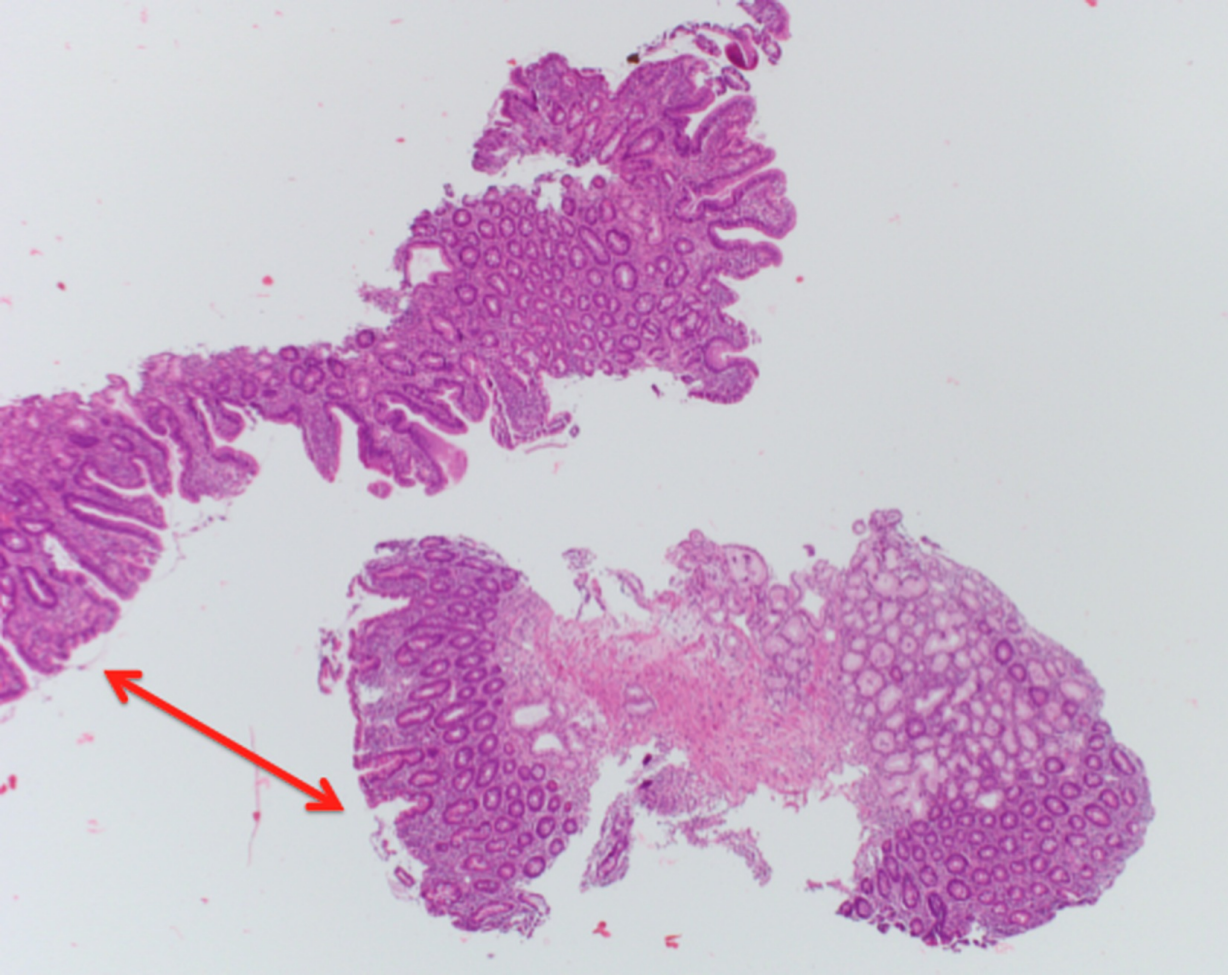
Higher magnification biopsy image (H&E stain) of the duodenum. Image showing variable flattening of the villi (red arrows).

**Figure 4:**
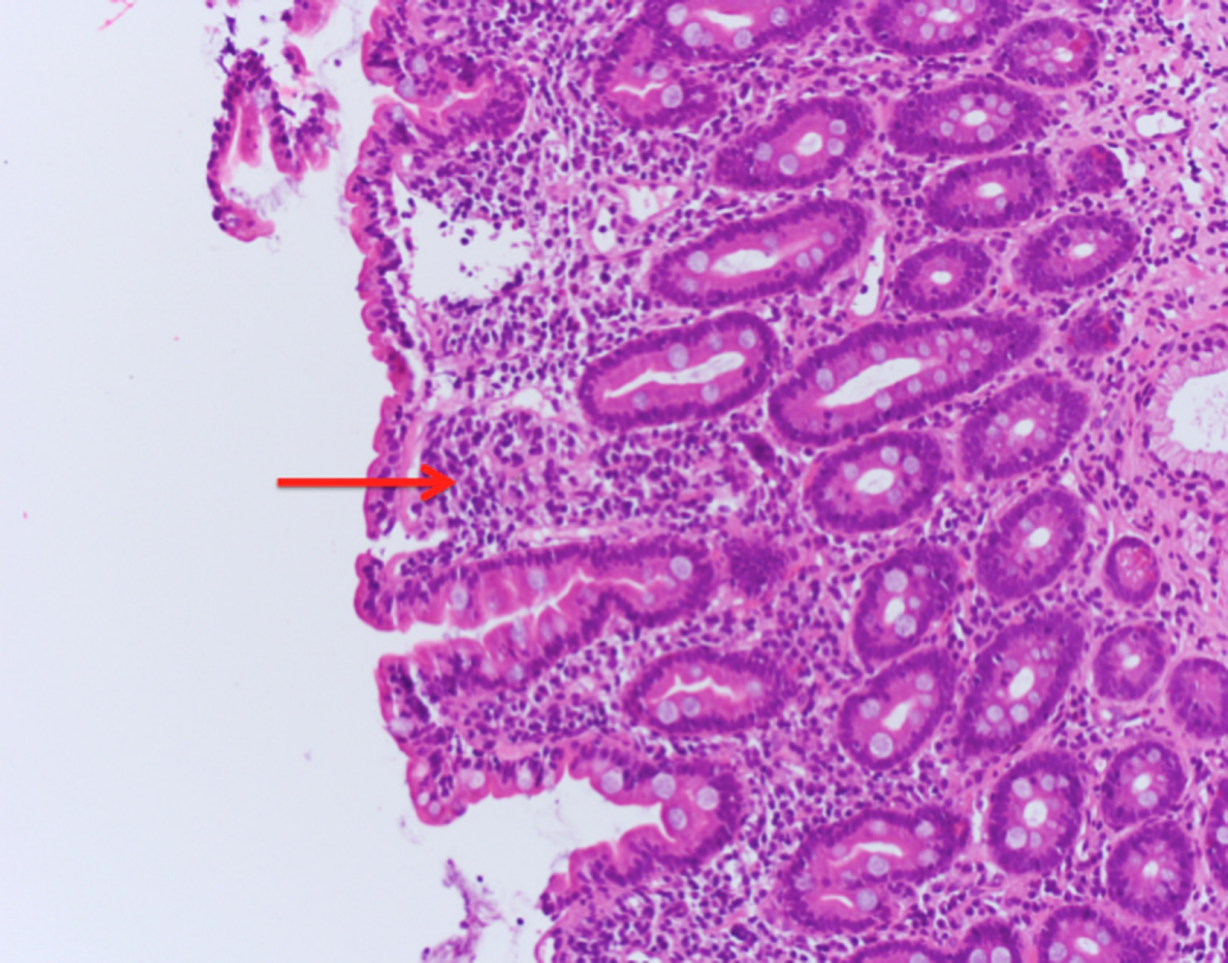
Higher magnification biopsy image (H&E stain) of the duodenum. Image showing lymphocyte infiltration (red arrow) and distention of the lamina propria, which is characteristic of celiac disease.

Findings from an upper gastrointestinal endoscopy were normal but duodenal biopsies showed flattening of the villi, Brunner's gland hyperplasia, lymphocytic infiltration, and distention of the lamina propria. These findings are consistent with celiac disease (Figures 2-4).

**Figure 5:**
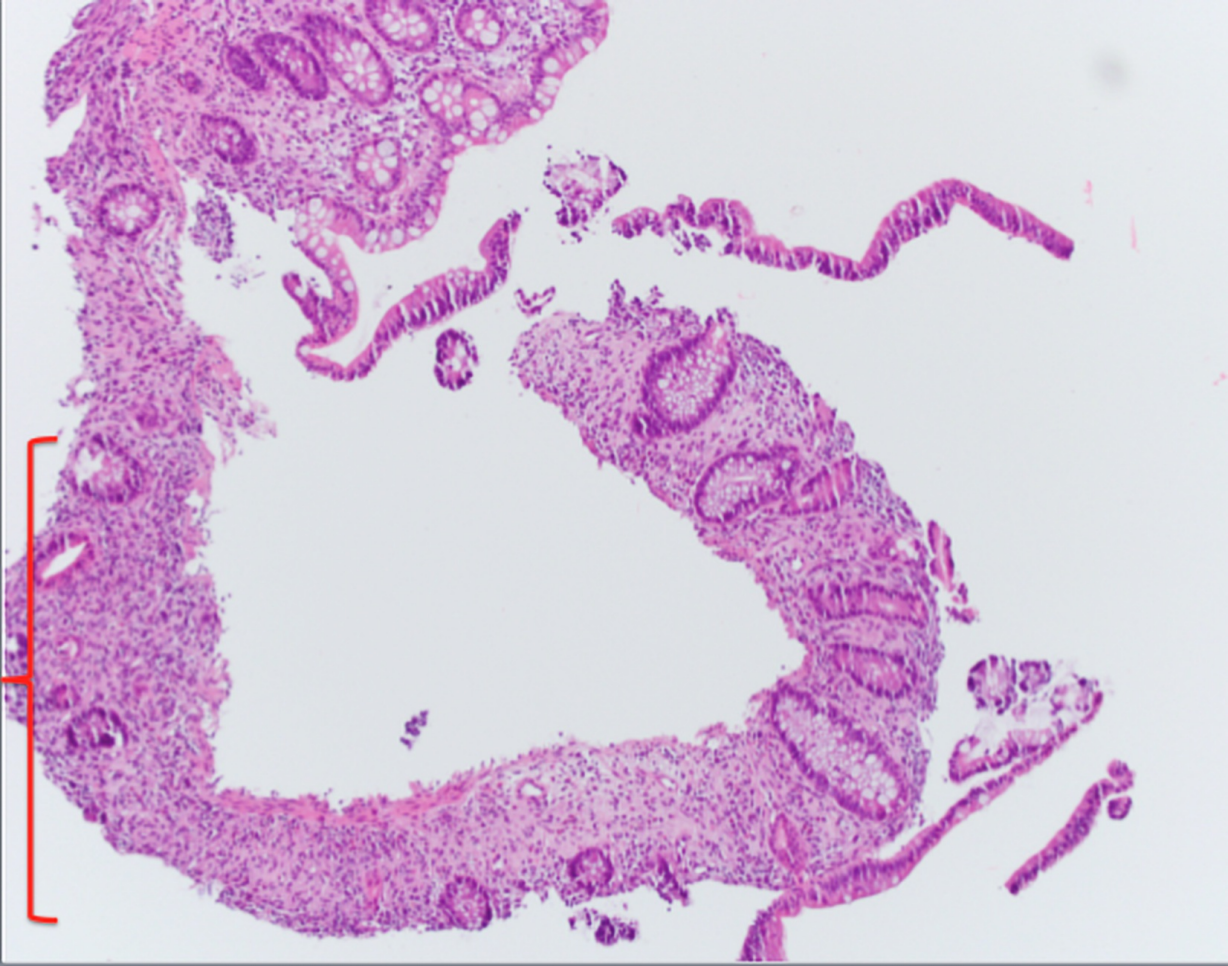
Biopsy image (H&E stain) of the cecum. Image showing detachment of the epithelium, fibrosis, and absence of glands (red margin), consistent with nonspecific inflammatory bowel disease.

In addition, biopsies from the cecum region showed detachment of the epithelium, absence of glands, and fibrosis consistent with nonspecific mild inflammatory bowel disease (Figure 5).

Serologic testing was positive for anti-transglutaminase antibodies, anti-endomysial antibodies, and anti-gliadin antibodies, which further confirmed the diagnosis of celiac disease. However, considering the colonoscopic findings (Figure 1) and cecum biopsy findings (Figure 5), the possibility of an underlying or evolving inflammatory bowel disease couldn't be ruled out. 

The patient was started on a gluten-free diet, resulting in resolution of diarrhea within one week. During the hospital course, the patient's laboratory workup also showed deficiency of vitamin A, D, and E. The patient was put on vitamin supplements and was discharged.

## Review

Celiac disease and nutritional deficiencies 

Celiac disease is characterized by malabsorption, weight loss, and vitamin and mineral deficiencies. Individuals diagnosed with CD are often found to have deficiencies in water-soluble vitamins or B vitamins as well as fat-soluble vitamins. In addition, individuals diagnosed with celiac disease also experience mineral deficiencies including deficiencies of calcium, copper, folate, and zinc. With the proximal small intestine being the most prominent affected organ in CD, deficiencies of water-soluble vitamins are expected as this is the primary location of absorption.

In 2001, Dahele and Ghosh determined that of the individuals in their cohort who went untreated for CD, 41% experienced anemia. Of those patients, 41% experienced B12 deficiency and 31% experienced folate deficiency [3]. In 2002, another study indicated that the B12 deficiency experienced by those with celiac disease was not due to autoimmune gastritis [4]. Although B12 is typically absorbed through the terminal ileum, the macrocytic anemia seen in untreated celiac disease patients is typically caused by folate deficiency [1]. In a 2007 study, Harper et al. determined that in their cohort, 31% of untreated patients with celiac disease developed iron deficiency anemia [5].

As for water-soluble vitamins, a study performed in 2008 determined that B2 levels were not compromised even in untreated patients with celiac disease [6]. In other studies, decreased pyridoxal phosphate was reported upon examination of duodenal mucosa of patients with celiac disease indicating B6 deficiency. Furthermore, vitamin B6 levels were decreased in children who had gone untreated for celiac disease. In summary, approximately one in seven patients with untreated celiac disease were determined to experience deficiency of water-soluble vitamins even with 20% of patients already being supplemented with over-the-counter vitamins and supplements [1].

Deficiencies of fat-soluble vitamins including vitamin A, D, and E were often observed in patients with untreated celiac disease. Vitamin A deficiency was seen equally in patients with both treated and untreated CD [7]. One of the studies found that 55% of patients with CD experienced vitamin D deficiency, which is often associated with osteomalacia [1,8]. A study performed by Hozyasz et al. determined that vitamin E was decreased in all patients with untreated CD, and that complications of this deficiency led several patients to experience neurological impairment [7]. Although uncommon, copper deficiencies have also been reported in patients with untreated CD and have produced neurological impairments [9]. Zinc deficiency was also common in patients with CD. Zinc deficiency is likely explained through increased endogenous loss of zinc instead of losses through zinc absorption [1].

In a study performed in 2013, the authors determined that 90% of patients with newly diagnosed CD experienced one or more of the nutritional deficiencies discussed. In addition, approximately half of these patients were deficient in two or more vitamins or minerals. Deficiencies seen in these patients were determined to be unrelated to the severity or duration of celiac disease. In addition, there were no statistically significant differences in vitamin or mineral deficiencies found between men and women with untreated CD [1].

It is unclear what definitive mechanism causes these vitamin or mineral deficiencies in patients with CD; even those individuals with mild CD were observed to have vitamin or mineral deficiencies. Nevertheless, individuals with CD should be counseled on dietary habits and means to reduce exacerbations of the disease. In addition, these patients should be supplemented with nutritional supplements when a deficiency is determined. These patients should be monitored consistently for progression or complications observed in these deficiencies [1].

Celiac disease and rheumatoid arthritis

In 2015, Lerner and Matthias determined that there was a significant amount of symptomatic and physiology overlap between CD and RA. Starting at the fundamental level, both CD and RA are autoimmune disorders that stereotypically affect certain aspects of the human body more so than other areas; RA affects the joints and CD affects the small intestine. At the genetic level, RA and CD have different predispositions, RA is related to human leukocyte antigen HLA DRB1 haplotypes and CD is related to HLA DQ2 and DQ8 haplotypes [10]. However, in a cross-sectional study by Shor et al. it was found that the autoantibodies most commonly found in CD, namely the immunoglobulin G anti-gliadin antibodies (IgG AGA), presented in twelve of the 186 patients with RA [11].

In addition to unique autoantibodies now being presented in similar autoimmune disorders, the inflammation present in each disorder is believed to be derived from similar causes. In a study by Cupi et al. it was found that the CD-related inflammation lead to a diminished clearance of apoptotic cells. Specifically, there were reduced mucosal levels of CD36, CD61, and TSP-1, which mediate phagocytosis of apoptotic bodies [12].

Celiac disease and sepsis

Sapone et al. in their study established that individuals with CD have increased permeability of the small intestine as well as impaired function of the mucosal barrier [13]. In 2016, Tjernberg et al. created a nationwide longitudinal registry-based study to establish the relationship between celiac disease and sepsis [14]. It was not established that individuals with CD were predisposed to becoming septic but rather that there was a mild increase in overall mortality should CD individuals become septic [14]. While a direct cause and effect link between CD and sepsis hasn’t been fully established, there is a correlation between hyposplenia in those with CD [15]. In the event that an individual is exposed to gluten over a long duration and is unable to tolerate it, they are at an increased risk of hyposplenia; however, if the individual is able to maintain a gluten-free diet spleen function may improve [15-16]. Due to a weaker spleen, CD individuals are at risk of increased mortality from infection by streptococcal, pneumococcal, and gram-negative bacteria. Due to their encapsulated nature, pneumococci, Haemophilus influenzae, and Haemophilus meningococci are of serious concern because the immune system relies on the spleen for removing such organisms [15].

Celiac disease and risk of non-Hodgkin's lymphoma

According to a study by Green et al., those with CD are at an increased risk of malignancy. However, the exact mechanism is unknown but is thought to be a combination of inflammatory cytokines, chronic antigenic stimulation, chronic inflammation, and increased permeability. Compared to the public, patients with CD, regardless of their diets, are nine times more likely to develop non-Hodgkin’s lymphoma (NHL) [17].

Similar findings were reported by Catassi et al. regarding an increased risk of non-Hodgkin’s lymphoma. The case control study performed by Catassi et al. demonstrated that not only was the risk of non-Hodgkin’s lymphoma increased, but also the risk of B and T cell enteropathy associated lymphoma. Six participants out of 653 had NHL and CD, three had a B type NHL and the remaining three had T cell type NHL. While the increased risk of lymphoma in those with CD was not as high as the study hypothesized, it was still deemed as a moderate risk factor for NHL [18].

Celiac disease and inflammatory bowel disease

There are not many studies published that document a deep relationship between celiac disease and inflammatory bowel disease (IBD); however, there are a few that do report the possibility of a relationship between celiac disease and inflammatory bowel disease. According to Giovanni et al., IBD could obfuscate the symptoms of celiac disease because they share common symptoms such as weight loss, abdomen pain, and diarrhea [19]. To add to the confusion, Culliford et al. reported three patients that presented with scalloping of the duodenal mucosal folds that were believed to be from villous atrophy; pathology that is commonly attributed to celiac disease. However, with histology confirmation and positive markers from Crohn’s disease with negative markers for celiac disease, the villous atrophy was attributed to the progression of the patient’s Crohn’s disease [20].

In order to determine if there was a possible association between celiac disease and IBD, Oxford et al. completed a retrospective study. It was found that the prevalence of IBD among those with celiac disease was higher than expected; compared to the general North American population, those diagnosed with celiac disease had a Crohn’s disease prevalence of 4.0% and ulcerative colitis prevalence of 3.2%. Compared with the same North American population, celiac disease was much less prevalent in those diagnosed with Crohn’s disease or ulcerative colitis, with a respective 1.27% and 1.04%. This suggests that for an individual suffering from celiac disease which is non-responsive toward a gluten-free diet, a workup for an underlying IBD should be completed [21].

Celiac disease and thyroid disorders

While there are theories that celiac disease and inflammatory bowel disorders are linked, there are thoughts that celiac disease is associated with other autoimmune disorders, namely diseases involving the thyroid. Sattar et al. completed a prospective study to establish if there was such an association between celiac disease and autoimmune thyroid diseases. It was found that there was an increased prevalence of 2.3% of autoimmune thyroid diseases in diagnosed celiac pediatric patients compared to the general pediatric United States population prevalence of only 0.3% to 1.25% [22].

The pathophysiology behind such an increase in prevalence could be explained by the prospective study of Ventura et al. that investigated antibodies as a possible cause. For two years, their study followed 90 patients with celiac disease and found an increase in prevalence of diabetes and thyroid related serum antibodies at 11.1% and 14.4%, respectively. The data obtained from the study suggests that gluten itself could be a reason for the increase in antibodies. Patients with celiac disease who consumed a diet with gluten were found to have increased levels of thyroid-related and antiphospholipid autoantibodies when compared to celiac disease patients who consumed a gluten-free diet. It was also found that when gluten was removed from the diet, the levels of autoantibodies would decrease. While the exact mechanism between gluten and the autoantibodies hasn’t been fully investigated yet, it is believed that if a gluten-free diet is started early enough, it can attempt to inhibit progression towards development of the autoantibodies [23].

Celiac disease and type 1 diabetes

While it appears that thyroid autoimmune disorders have an association with celiac disease, type 1 diabetes (T1D) might have a greater prevalence in the celiac disease population. Compared to earlier stated prevalence percentages, the study completed by Camarca et al. has documented that amongst the celiac disease population, T1D had a prevalence ranging from 4.4% to 11.1% versus the 0.5% T1D prevalence of the general population. The reasoning behind the increased prevalence is due to the shared HLA genotype background; DR3-DQ2 for celiac disease, DR3-DQ2 and DR4-DQ8 for T1D [24].

In addition to similar genotype backgrounds, the research performed by Fasano et al. believes that there is another process by which celiac disease and type 1 diabetes are similar. The protein zonulin, an intestinal lumen tight junction regulator, was found to affect those with celiac disease and those with type 1 diabetes in similar methods. Zonulin was found to be upregulated during the acute phase of celiac disease, which would cause an increase in the permeability of the intestinal lumen [25]. The study performed by Visser et al. established that gliadin can release preformed zonulin and cause an increase in intestinal permeability. In the event of a gluten-free diet, zonulin levels and intestinal permeability decreased [26].

## Conclusions

In this article we highlighted associations between celiac disease and other conditions, which often go unnoticed in a clinical setting. Though all of the studies conducted consisted of relatively small groups of patients, the outcomes are still concerning. Therefore, validating these results through larger cohort studies is needed.
